# Comparative analysis of racial differences in breast tumor microbiome

**DOI:** 10.1038/s41598-020-71102-x

**Published:** 2020-08-24

**Authors:** Srikantha Thyagarajan, Yan Zhang, Santosh Thapa, Michael S. Allen, Nicole Phillips, Pankaj Chaudhary, Meghana V. Kashyap, Jamboor K. Vishwanatha

**Affiliations:** 1 Department of Microbiology, Immunology, and Genetics, University of North Texas Health Science Center, Fort Worth, TX 76107 USA; 2 Texas Center for Health Disparities, University of North Texas Health Science Center, Fort Worth, TX 76107 USA; 3 Department of Pathology, Texas Children’s Microbiome Center, Texas Children’s Hospital, Houston, TX 77030 USA; 4 Department of Pathology and Immunology, Baylor College of Medicine, Houston, TX USA; 5 University of Nebraska Medical Center, Omaha, NE USA

**Keywords:** Cancer, Microbiology, Molecular biology, Pathogenesis

## Abstract

Studies have demonstrated that environmental, host genetic, and socioeconomic factors influence the breast cancer prevalence landscape with a far-reaching influence on racial disparity to subtypes of breast cancer. To understand whether breast tissue harbors race-specific microbiota, we performed 16S rRNA gene-based sequencing of retrospective tumor and matched normal tissue adjacent to tumor (NAT) samples collected from Black non-Hispanic (BNH) and White non-Hispanic (WNH) women. Analysis of Triple Negative Breast cancer (TNBC) and Triple Positive Breast Cancer (TPBC) tissues for microbiota composition revealed significant differences in relative abundance of specific taxa at both phylum and genus levels between WNH and BNH women cohorts. Our main findings are that microbial diversity as measured by Shannon index was significantly lower in BNH TNBC tumor tissue as compared to matched NAT zone. In contrast, the WNH cohort had an inverse pattern for the Shannon index, when TNBC tumor tissue was compared to the matched NAT. Unweighted Principle Coordinates Analysis (PCoA) revealed a distinct clustering of tumor and NAT microbiota in both BNH and WNH cohorts.

## Introduction

TNBC, a breast cancer disparity with an especially aggressive subtype, is more prevalent in Black non-Hispanic (BNH) than in White non-Hispanic (WNH) women^1,2^. Breast cancer is highly heterogenous with multiple subtypes where each subtype initiates and develops as a result of defective functioning of regulatory and signaling pathways^3,4^. BNH women manifest breast cancer disparity as higher mortality rate, poorer prognosis due to higher relapse, and higher incidence rates under age 45 than do WNH^5,6^. BNH women are approximately twice as likely to be diagnosed with highly aggressive estrogen receptor negative (ER-), estrogen and progesterone receptor negative and Her2 positive, or TNBC subtypes of basal-type breast cancer^7–9^. Several studies have shown that the paired normal tissue adjacent to tumor (NAT) as compared to tumor tissue did not differ in microbiota richness as reflected by the number of operational taxonomic units (OTUs), but differed in evenness as reflected by the abundance of specific species of bacteria in normal vs tumor zone^10–12^. Treatment of rats with *Lactobacillus casei* has been shown to reduce adjuvant-induced arthritis and increased abundance of other *Lactobacillus* species^13^. These findings are of interest as the antiproliferative activity of *Lactobacillus crispatus* and *Lactobacillus acidophilus* was found to be associated with health status of breast cancer cells^14^. The beneficial influence of breast feeding and breast milk in establishing the infant gut microbiota and health during adult life has been well documented in many studies^15,16^. A limited number of recent studies also demonstrated an association between compositional differences of microbial species between healthy and breast tumor tissues including unique bacterial microbiome signatures between different subtypes of breast cancer^12,17,18^.

Association of specific microbiome profiles with somatic genome SNP from colorectal tumor cells and matched normal tissue^19^ suggest a role of host and tumor genetic backgrounds in microbiota structure. Among biological factors, epigenome-driven microbiota profile has been implicated in the etiology of several kinds of cancer including the association with breast cancer^20^. Several studies on gut and oral microbiota have demonstrated that association of distinct microbiota in different geographical and regional differences in human population cohorts adopting different lifestyle/occupation or socioeconomic status (SES)^21,22^. Bioinformatic analysis of published gut microbiota datasets showed association of distinct bacterial species among Caucasian, African American, Pacific islanders and Hispanic racial/ethnic groups^23^. A recent study demonstrated that racial differences existed in breast microbiota richness and specific bacterial taxa between WNH vs BNH women breast tumor tissue and adjacent normal tissue, which were derived from a biorepository representing the population cohorts encompassing the Black Belt Region of the southeastern U.S.^24^. In this study, we refer Caucasian as White non-Hispanic (WNH) and African American as Black non-Hispanic (BNH).

Therefore, the intent of this study was two-fold: first, to address whether racial differences in microbiota are associated with archived retrospective breast tissue of tumor and adjacent normal tissue among BNH and WNH women population cohorts representing New England, USA; second, to determine if racial ancestry is strongly associated with microbiome profiles irrespective of cancer subtypes. This information will lay the crucial foundation to understand the extent of influence of geography, race, and ancestry/genetic background on microbiota profiles and increased incidence of aggressive TNBC subtypes within African American (BNH) populations.

## Results

### Breast tissue sample collection traits and specimen category

The deidentified frozen breast tissue samples were collected retrospectively from the biorepository of the Fox Chase Cancer Center, Philadelphia, PA (Table S1). All the breast cancer patients were residing in the New England region of USA. The age of the women at the time of breast tissue collection ranged between 27–78 years. The women were not subjected to antibiotic regimen prior to the collection of tissue samples. In addition, none of the patients had undergone radiation treatment. For the first set of microbiome analysis, we selected tumor and matched NAT. For the analysis, 6 TNBC WNH and 7 TNBC BNH, 7 TPBC WNH and 3 TPBC BNH were chosen. To verify the microbial diversity results, we analyzed a second set of 10 WNH TNBC samples of tumor and matched NAT.

### Ancestry of breast cancer subjects

**Figure 1:**
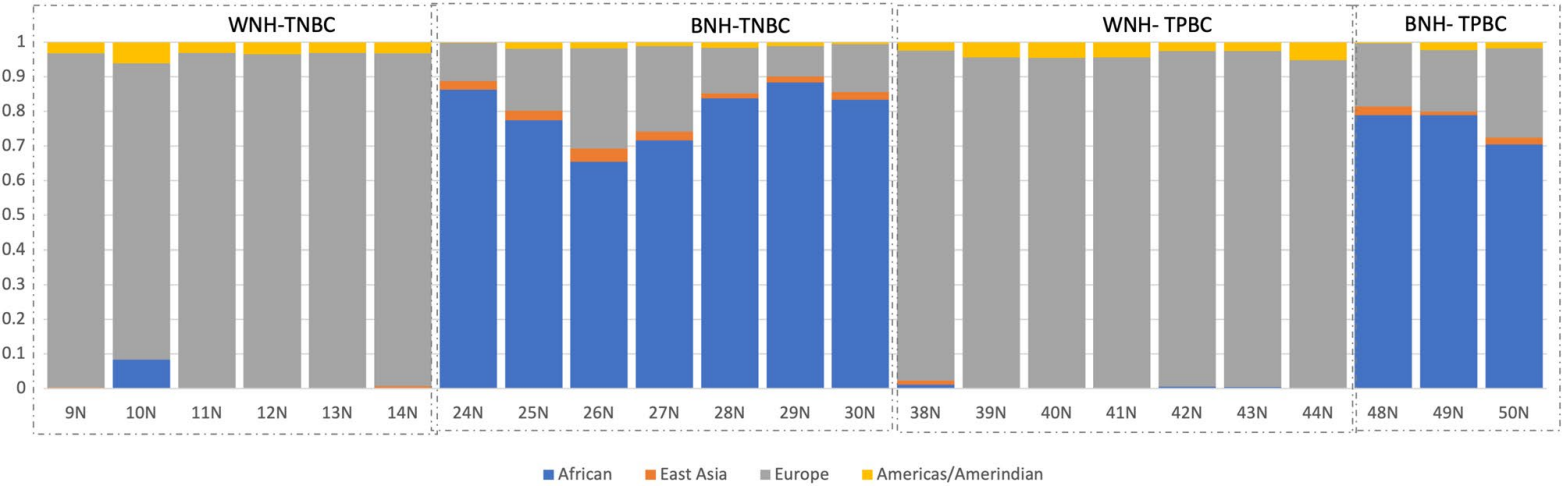
Ancestry Analysis. The genetic ancestry of both tumor and normal tissue samples were analyzed using an Illumina Infinium Global Screening Array, and the data were analyzed using the methods as described in the text. The decoded Matched Normal samples are labelled as symbol N following the sample number in the study. WNH and BNH represents White non-Hispanic and Black non-Hispanic racial cohorts respectively.

All tissue samples yielded reproducible ancestral assessment in individuals of primarily one ancestral background, confirming genetic ancestry concordance with self-described race/ethnicity (Fig. 1). As expected, admixture was detected in the BNH individuals, with the largest ancestral component deriving from African descent. WNH individuals showed a higher degree of homogenous ancestry, with the primary ancestral component indicated being European.

### Microbiota composition in breast tissue of cancer patients

**Figure 2:**
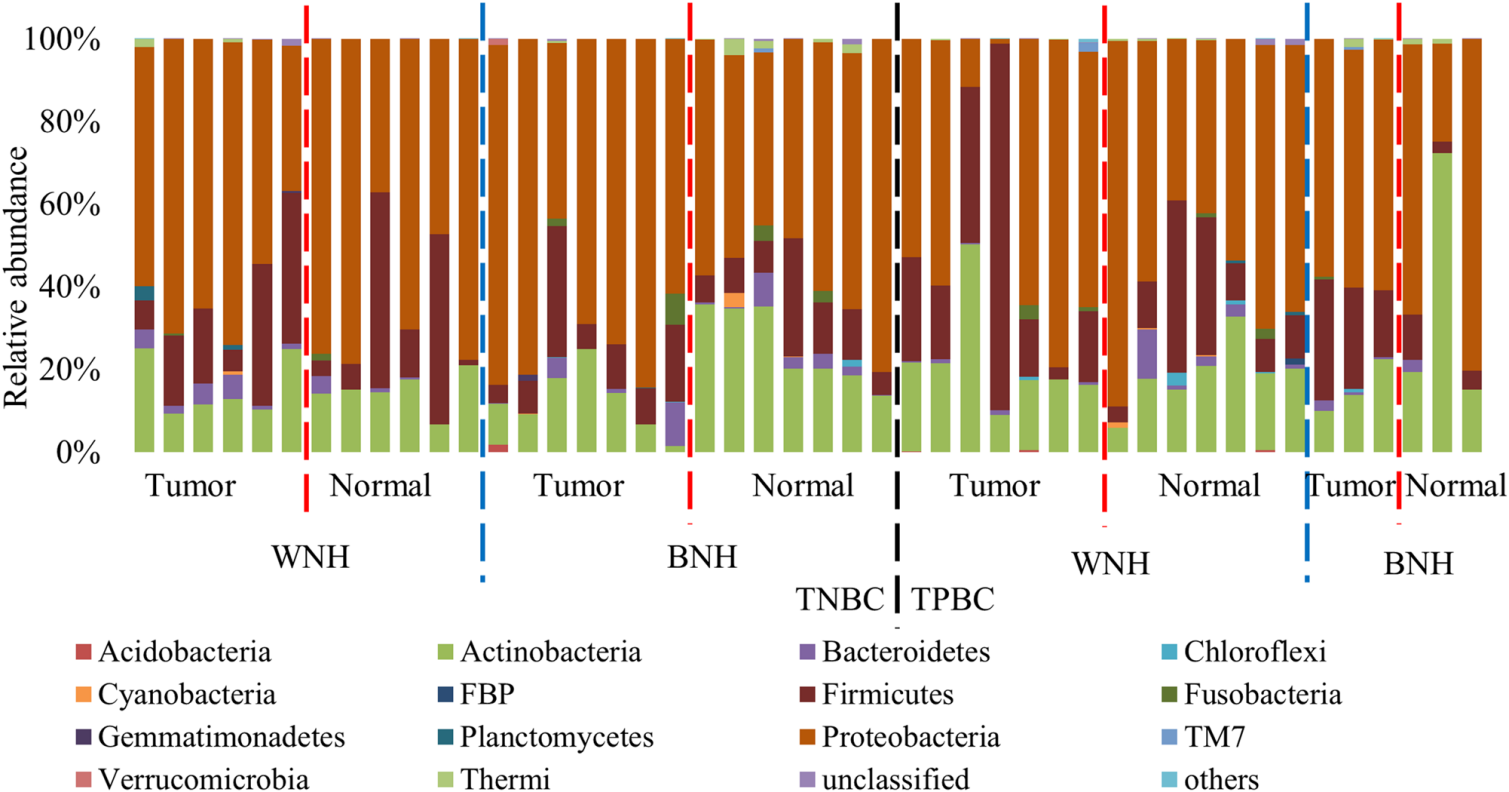
Normalized bar plot of bacterial community composition in tumor and normal tissues at the phylum level. Phyla with > 1% relative abundance are shown. TNBC, triple negative breast cancer; TPBC, triple positive breast cancer; BNH, Black non-Hispanic; WNH, White non-Hispanic.

**Figure 3:**
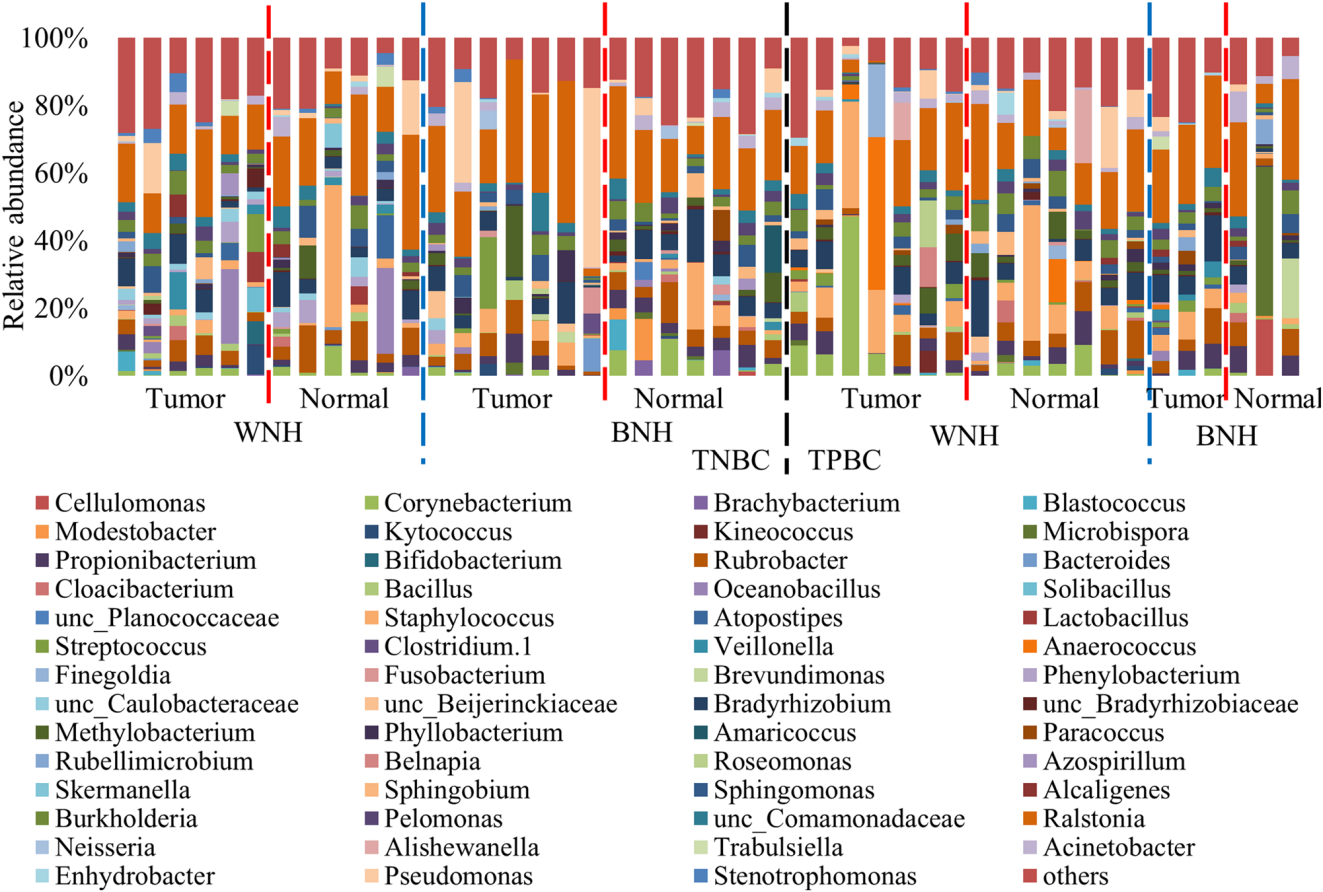
Normalized bar plot of bacterial community composition in tumor and normal tissues at the genus level. Genera with > 1% relative abundance are shown. TNBC, triple negative breast cancer; TPBC, triple positive breast cancer; BNH, Black non-Hispanic; WNH, White non-Hispanic.

Comparison of the microbiota in tumor and adjacent normal breast tissues from 6 (excluding two admixed outliers based on ancestry analysis) WNH, 7 BNH TNBC patients, as well as 7 WNH, 3 BNH TPBC patients showed a total of 20 bacterial phyla and 419 genera were found in the breast tissue of cancer patients (Table S2). Microbial abundance at different phylogenetic levels of the above 46 samples (a set) and 20 samples from the second set (b set) are shown in Table S2. Across all the breast tissue samples, estimation of relative abundance revealed that the most dominant bacterial population was Proteobacteria (59.4% ± 18.6%), followed by Actinobacteria (19.1% ± 12.0%), Firmicutes (17.7% ± 16.5%), and Bacteroidetes (1.9% ± 2.8%) (Fig. 2). The top 5 most abundant bacterial genera were *Ralstonia*, *Staphylococcus*, unclassified Bradyrhizobiaceae, *Rubrobacter* and *Pseudomonas*. The bacterium *Ralstonia* (19.1% ± 9.0%) was the most dominant bacterial genus prevalent in all the tumor and normal tissues (Fig. 3). The bacterial genus *Staphylococcus* (6.4% ± 9.4%) was the second dominant bacterium detected in 21 out of 23 NAT and 22 out of 23 tumor breast tissues (Fig. 3). The bacterial genera unclassified Bradyrhizobiaceae (5.5% ± 4.0%) and *Rubrobacter* (5.4% ± 3.3%) were also observed in all the breast tissues including tumor and adjacent normal zone (Fig. 3). *Pseudomonas* (4.1% ± 9.5%) was prevalent in 17 out of 23 normal and 17 out of 23 tumor breast tissues (Fig. 3).

### Microbiota diversity and community composition in tumor tissues was different from normal tissues

**Figure 4:**
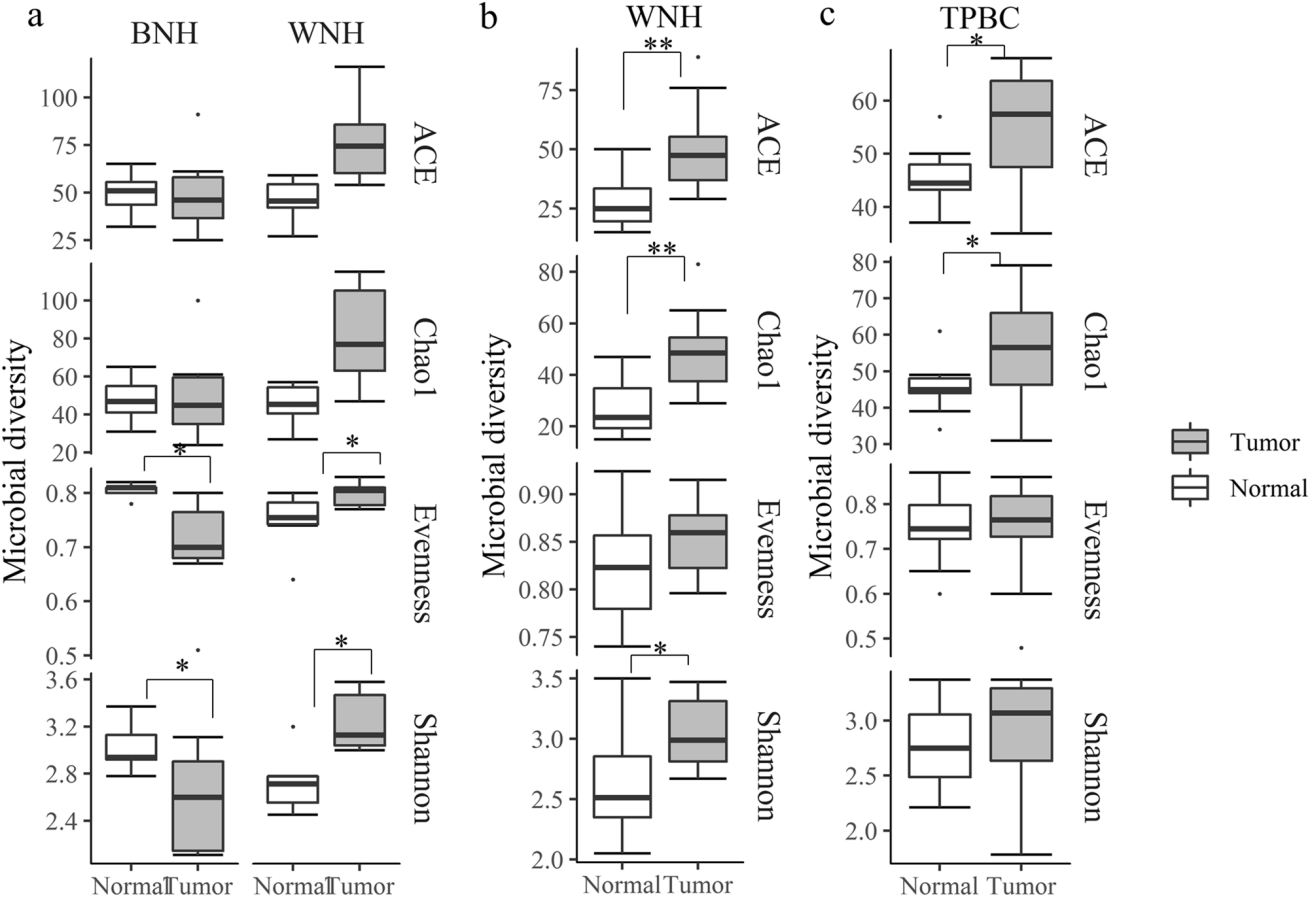
Bacterial diversity in tumor and adjacent normal tissue of (**a**) TNBC from WNH (n = 6) and BNH (n = 7); (**b**) TNBC WNH 2nd set (n = 10) and (**c**) TPBC pool (n = 10) cancer patient samples. Dots represent outliers; *p < 0.05; **p < 0.01, Wilcoxon Signed Rank Test.

The microbial diversity differences between tumor and normal tissue showed different patterns in WNH and BNH populations. For the BNH population with TNBC, the Shannon diversity (*p* = 0.05) and evenness (*p* = 0.04) in tumor tissues were significantly lower than the NAT tissue (Fig. 4a). In contrast, for the WNH population with TNBC, the Shannon diversity (*p* = 0.05) and evenness (*p* = 0.04) of tumor tissue were significantly higher than the NAT. TNBC WNH population also had higher richness (ACE *p* = 0.06; Chao1 *p* = 0.06) in tumor than in normal tissue, although not statistically significant (likely due to sample size and sample heterogeneity). In order to confirm the results of the reversed pattern of alpha diversity metrics, we sequenced an additional 10 breast tumor tissues from WNH TNBC tissues along with NAT. The results revealed that microbial diversity indices showed the same pattern indicating higher Shannon diversity (*p* = 0.04) and richness (ACE *p* = 0.004; Chao1 *p* = 0.006) in WNH tumor samples (Fig. 4b).

In TPBC patients, tumor tissue showed significantly higher alpha diversity metrics (ACE (*p* = 0.04) and Chao1 (*p* = 0.05) richness) than normal tissue (Fig. 4c). The Shannon diversity and evenness between tumor and NAT did not show significant differences, possibly due to high inter-individual heterogeneity.

**Figure 5:**
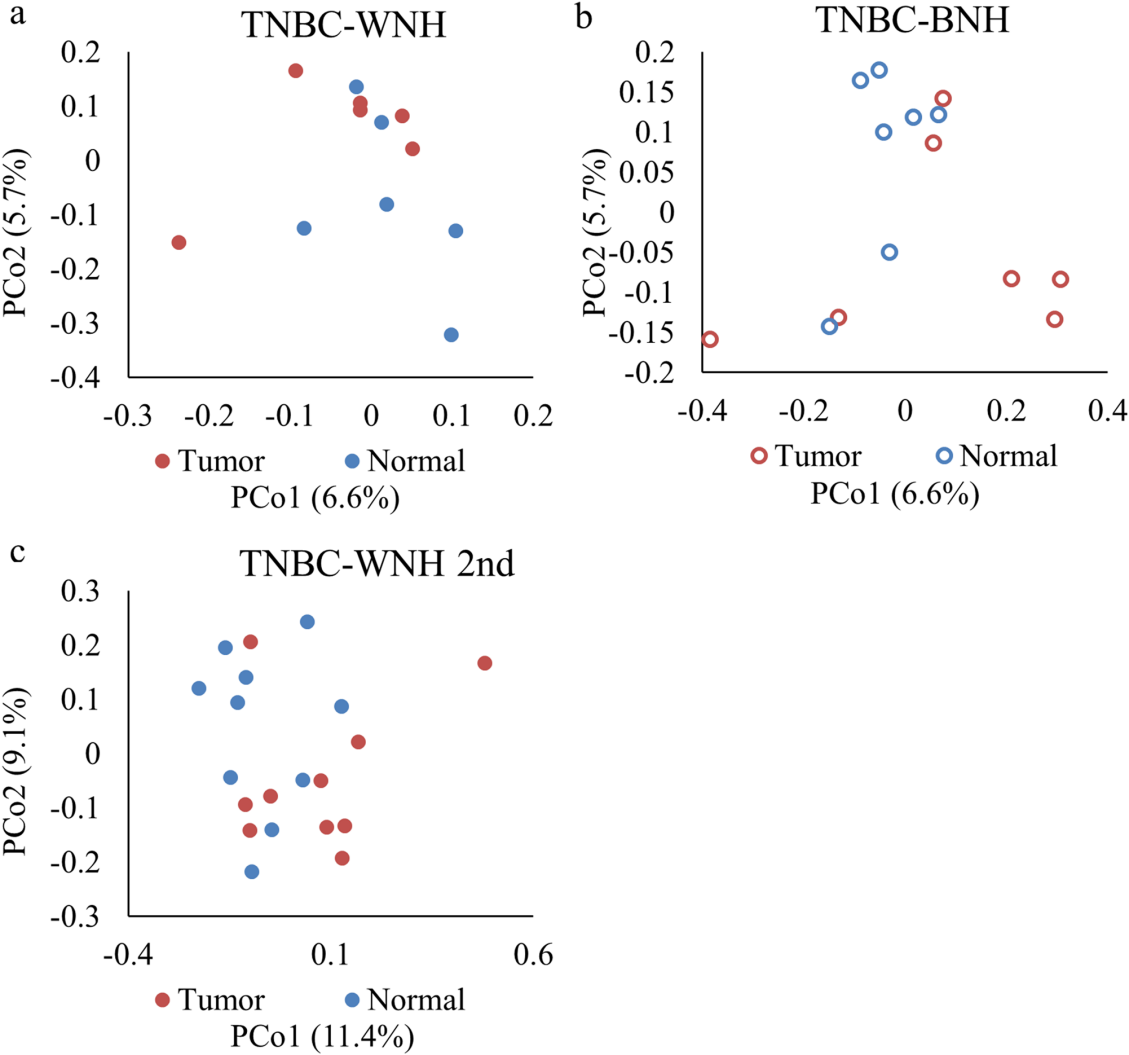
Principal coordinate analysis (PCoA) based on unweighted UniFrac distances showing clustering of tumor vs normal tissues for TNBC patients. TNBC-WNH(n = 6); TNBC-BNH (n = 7); TNBC-WNH 2nd (n = 10) refers to second set of samples.

Differences in microbial community structure between normal and tumor tissue were observed in TNBC patients using principal coordinate analysis (PCoA) ordination of unweighted UniFrac distances (Fig. 5). The unweighted PCoA plots showed different microbial communities between tumor and normal tissue in TNBC patients for both WNH (AMOVA *p* = 0.02) and BNH (AMOVA *p* = 0.07) groups (Fig. 5a and b). The PCoA based on weighted UniFrac distances did not reveal microbial community difference between tumor and normal tissue in TNBC patients. In the second set of TNBC WNH patients, the unweighted UniFrac PCoA analysis revealed some degree of distinct clustering of microbiota in tumor and NAT (AMOVA *p* = 0.06) (Fig. 5c).

**Figure 6:**
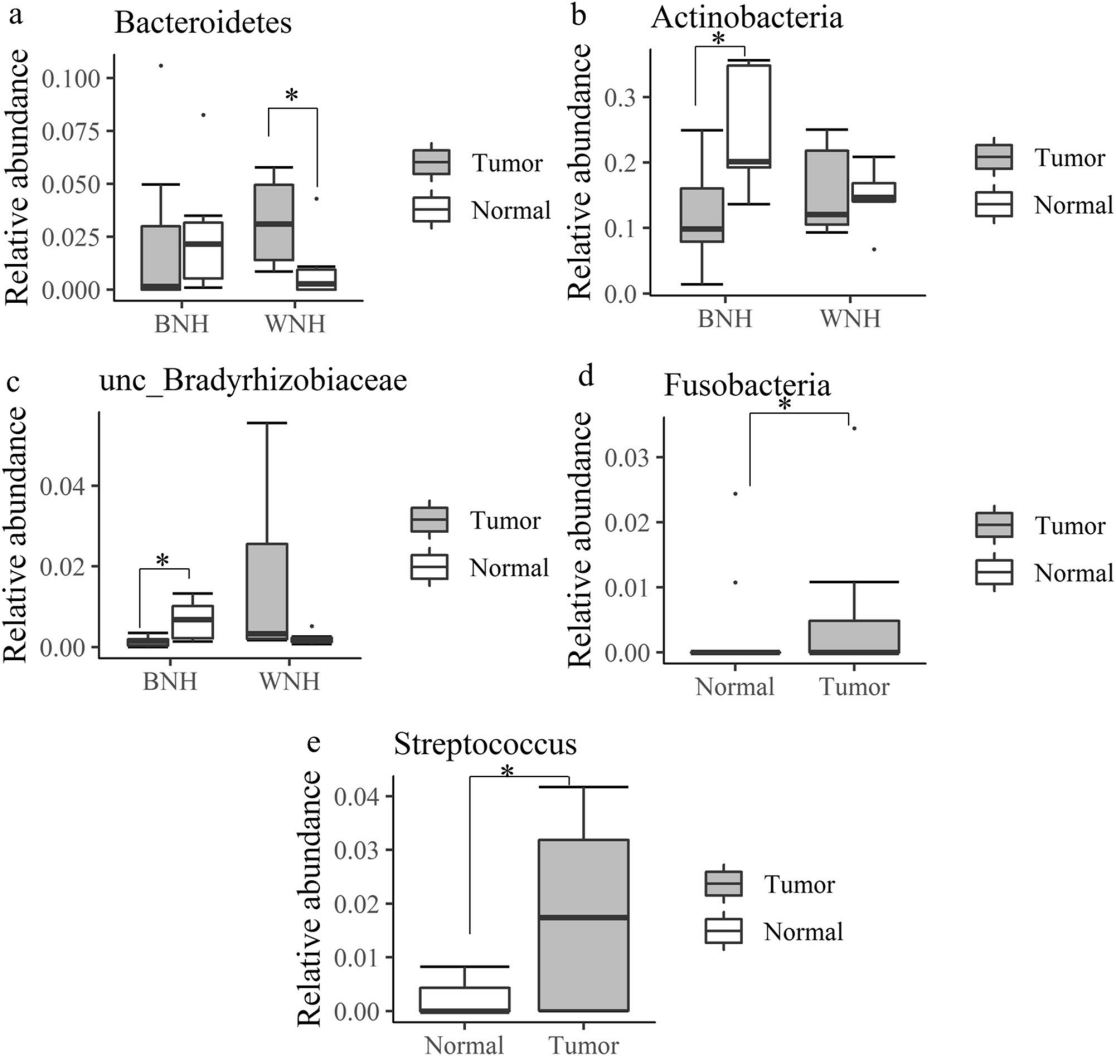
Microbiota relative abundance at phylum (**a**, **b**, **d**) and genus (**c**,**e**) levels showed differences between tumor and normal tissue in both TNBC and TPBC patients using Wilcoxon Signed Rank Test. (**a**–**c**) TNBC WNH (n = 6) and TNBC BNH (n = 7); (**d**, **e**) TPBC (n = 10). Dots indicate outliers; * *p* < 0.05.

To determine microbial differences between tumor and the normal tissue, we performed Wilcoxon Signed Rank Test with abundant bacterial phyla (> 1%) and genera (> 5%). The Wilcoxon Signed Rank test for microbiota composition revealed that in TNBC WNH patients, the phylum Bacteroidetes in tumor tissue was significantly higher than in NAT tissue (*p* = 0.03) (Fig. 6a). For TNBC BNH patients, the phylum Actinobacteria (*p* = 0.03) and unclassified genus of unclassified Bradyrhizobiaceae (*p* = 0.03) in tumor tissue were significantly lower than in normal tissue (Fig. 6b, c). For TPBC patients, at plylum level *Fusobacteria* (*p* = 0.01) showed higher abundance in the tumor tissue as compared to normal tissue. At genus level, *Streptococcus* (*p* = 0.03) showed the same trend(Fig. 6d, e).

#### WNH and BNH TNBC patients showed different microbiota diversity and community composition

The relative abundance of Bacteroidetes, showed a variable pattern in tumor vs normal breast tissue in both WNH and BNH groups. There was inter-individual heterogeneity in relative abundance of Bacteroidetes in both tumor and normal tissue within BNH samples (Fig. 6a). In contrast, the WNH group showed significantly lower variation and relative abundance in normal tissue as compared to tumor tissue (Fig. 6a).

**Figure 7:**
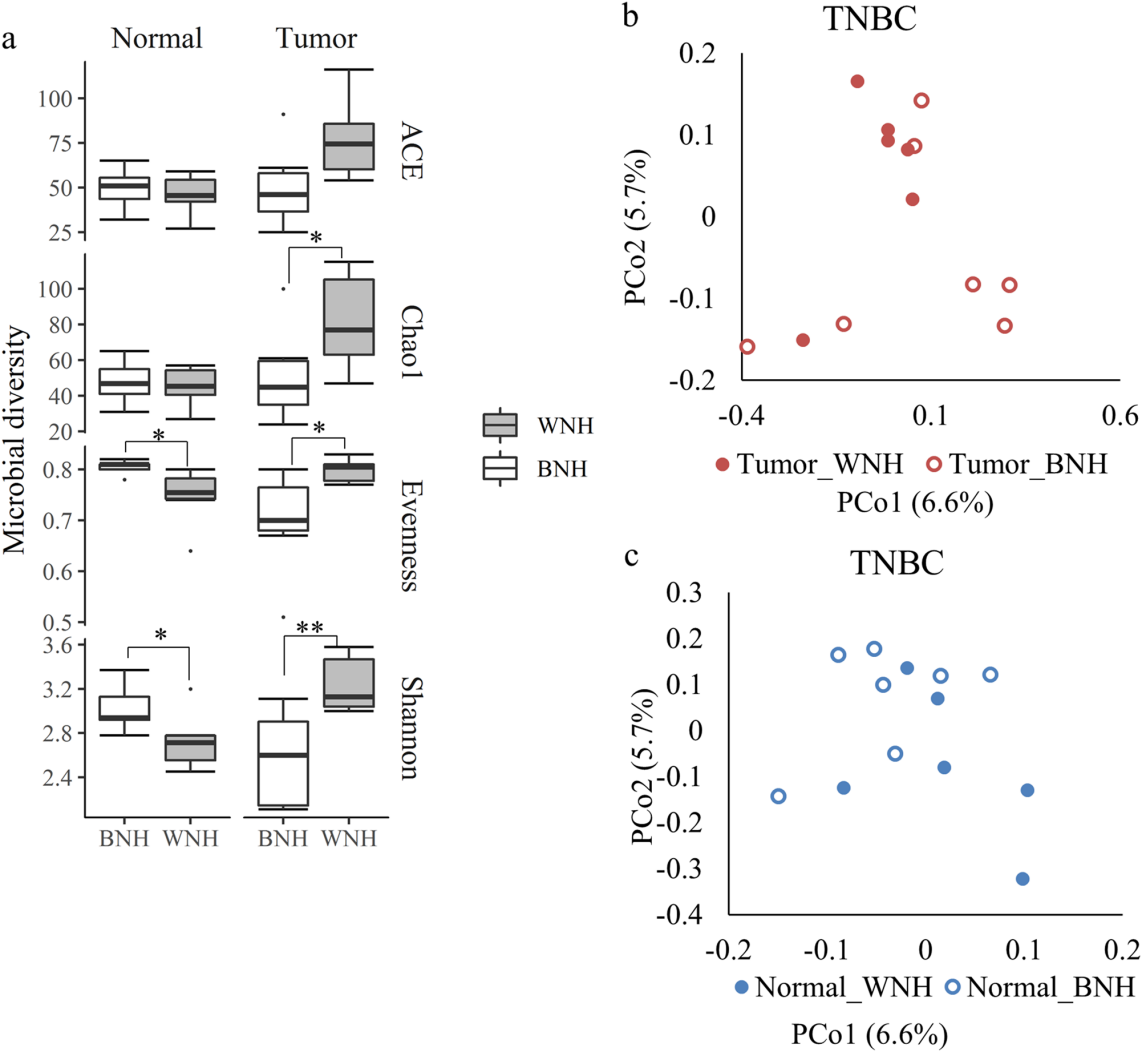
Comparison of microbiota of BNH and WNH in TNBC cancer patients. (**a**) bacterial community diversity and evenness; (**b**, **c**) Principal coordinate analysis (PCoA) based on unweighted UniFrac distances of (**b**) tumor and (**c**) normal adjacent tissues from WNH (n = 6) and BNH (n = 7) patient samples. Dots indicate outliers; *p < 0.05; **p < 0.01 determined by Wilcoxon Rank Sum Test.

We found microbiota compositional differences between WNH and BNH racial groups. The matched normal tissue of TNBC in BNH cohort showed a significantly higher Shannon diversity (*p* = 0.03) and evenness (*p* = 0.01) than the WNH (Fig. 7a), but very similar microbial richness as measured by both ACE and Chao1 metrices. In contrast, in TNBC, WNH showed significantly higher microbial diversity (Shannon *p* = 0.008, Chao1 *p* = 0.05) and evenness (*p* = 0.01) than the BNH (Fig. 7a). PCoA based on unweighted UniFrac distances showed that TNBC WNH and BNH had significantly different microbial communities for both tumor (AMOVA *p* = 0.05) and normal tissues (AMOVA *p* = 0.007) (Fig. 7b, c).

**Figure 8:**
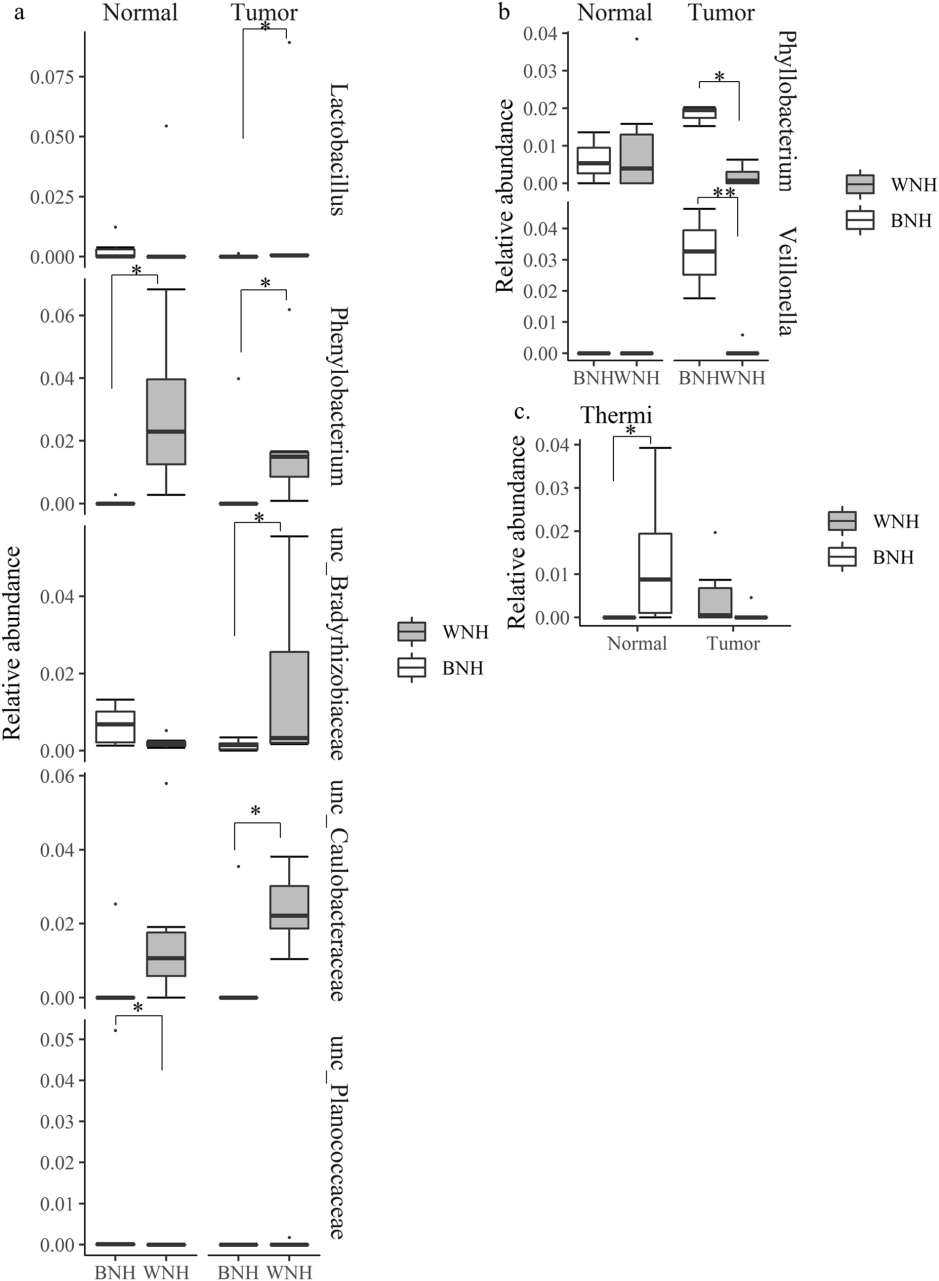
Relative abundance of taxa showed differences between BNH and WNH at the genus (**a**, **b**) and phylum (**c**) levels in TNBC (**a**, **c**) and TPBC (**b**) patients. BNH (n = 7), WNH (n = 6) Dots are outliers. *p < 0.05, **p s< 0.01, Wilcoxon Rank Sum Test.

To determine the microbial population differences between BNH and WNH samples, we performed a Wilcoxon Rank Sum Test with selected abundant bacterial phyla (> 1%) and genera (> 5%). At the genus level, several genera showed significant differences between BNH and WNH populations in both tumor and normal tissue. TNBC WNH patients had higher abundance of *Phenylobacterium* than BNH in both normal (*p* = 0.003) and tumor tissues (*p* = 0.02) (Fig. 8a). TNBC WNH patients had higher abundance of *Lactobacillus* (*p* = 0.02), in two unclassified genera within the families Caulobacteraceae (*p* = 0.02) and Bradyrhizobiaceae *(p* = 0.03) than BNH in tumor tissue. The normal tissue from TNBC BNH patients had a higher abundance of unclassified genus of Planococcaceae than WNH (*p* = 0.04) normal tissue, but not in normal tissue. At the phylum level, Thermi showed higher relative abundance in normal tissue and lower relative abundance in tumor tissue of TNBC BNH patients compared with WNH patients (*p* = 0.02) (Fig. 8c). For TPBC patients, no significant taxon difference between WNH and BNH was detected in normal tissue. However, TPBC tumor tissue showed higher abundance of *Veillonella* (*p* = 0.01) and *Phyllobacterium* (*p* = 0.02) in BNH than WNH tissues (Fig. 8b). No statistically significant microbial diversity or community differences were detected between WNH and BNH with TPBC normal tissue.

## Discussion

A significant health disparity exists among racial/ethnic populations in the US with African American ancestry in rates of TNBC^1,2^. Several studies have implicated a multitude of unique mechanisms such as apoptosis^26^, race-specific tumor environment^27^, somatic mutation landscape^28^ and mitochondrial function^29^ as mediators of health disparities and influenced by low socioeconomic status, psychosocial stress, life style and genetic ancestry. However, these mechanisms do not fully account for TNBC breast cancer disparity in African American populations. One of the biological factors suspected to play role in this health disparity is the microbiome^30^.

The analysis of microbiota composition showed a significantly lower microbiota evenness and Shannon index in TNBC BNH tumor as compared to adjacent normal tissue, as well as alterations in the proportions of specific taxa. In contrast, WNH tumor showed the opposite scenario, with a higher evenness/Shannon index in tumor tissue compared to normal tissue. Smith et al. observed a higher microbial diversity and richness in the adjacent normal pair than in the normal, and tumor tissue^24^. However they did not perform paired analyses between tumor tissue and the paired adjacent normal tissue. In addition, their data include both WNH and BNH. The majority of their adjacent normal tissue were from WNH with 2 samples from BNH. To confirm our results we sequenced a second set of WNH samples and performed a paired comparison between the tumor and adjacent normal tissue. The microbiome analysis of the second set of WNH breast tumor/matched normal tissue showed a similar pattern to we what observed from the first set. The reason for reverse pattern in diversity could be due to the compositional differences of beneficial vs pathogenic species as demonstrated in a previous study between native African and migrated African American populations on microbiota profiles with beneficial vs CRC carcinogenic inducing metabolites^31^. This unique finding may be due to differential abundance of specific microbial taxa among ethnic groups, which in turn may influence TNBC onset/prevalence, prognosis and survival differences between BNH and WNH groups. Interestingly, TPBC microbiome analysis showed an increase in microbial richness component irrespective of race, although the evenness component remained very similar between tumor and adjacent normal tissue. The TPBC microbiota data also indicate that microbial metabolism by estrogenic microbes may trigger tumorigenesis^32^, The breast tumor microbiota studies to date^33,34^ have shown that the abundance ratio among specific microbial members could be detected among ethnicities. For example, Canadian and Irish derived breast tissues showed distinct microbiota signatures^33^. In another study a change in evenness alone, which is usually associated with a change in abundance of specific microbial members like *Methylobacterium* and *Sphingomonas* was associated with breast tumor state^10^.

The analysis of microbial composition revealed that the highly abundant phylum Actinobacteria showed lower abundance in tumor in comparison to normal BNH of TNBC. The lower abundant phylum Bacteroidetes showed similar abundance in both tumor and normal tissues of TNBC BNH, but higher abundance in WNH tumor associated tissue and with high interindividual variability. Bacteroidetes represents one of the key anaerobic commensals in the gut with flexibility to become pathogenic under unfavorable conditions in the host^35^. The Firmicutes to Bacteroidetes (F/B) ratio is regarded to be of importance in human gut microbiota composition and increase in the ratio has been directly correlated to obesity^36,37^. However, the similar F/B ratio in both BNH and WNH tumors and the wide difference in F/B ratio between BNH and WNH normal tissues observed in this study will require further study to fully understand the ramifications in breast cancer and health disparities. The phylum Actinobacteria with significantly lower abundance in BNH tumors, suggests alterations in evenness. This may be due to possible changes in ratios between classic beneficial microbes*, Bifidobacterium* and *Streptomyces,* and putatively pathogenic members such as *Propionibacterium, Corynebacterium,* etc.^38^*.* A recent meta-analysis of the published breast cancer microbiome databases using One Codex revealed the prevalence of significant evenness differences between the majority of microbial groups including Bacteroidetes and Actinobacteria rather than richness differences between normal and disease states^34^*.* Future studies should focus on both high resolution analysis using shot-gun metagenomics to identify microbial phenotypes and host genetic signatures to develop effective personalized therapeutic strategies. A large scale in silico analysis of gut microbiome data sets has shown the existence of microbiota profiles unique to four ethnic groups that included both BNH and WNH^23^. In that study, BNH showed the lowest gut microbiota evenness and richness as measured by Shannon index. The extent to which this impacts the breast microbiome in tumor and healthy tissues remains to be elucidated. In another recent study on the breast microbiome, *Ralstonia* was shown to be abundant in BNH tumors, while the family Xanthomonadceae was abundant in WNH tumors^24^. In contrast, our study showed that *Ralstonia* was the dominant genus with no significant differences in abundance between WNH vs BNH or tumor vs normal tissues. One genus of unclassified Bradyrhizobiaceae showed a significant reduction in abundance and very low interindividual variability in BNH tumor as compared to high interindividual variability and higher abundance in normal tissue. Interestingly, the abundance trend was opposite in tumor and normal tissues in the WNH cohort. Since most studied members of this genus are nitrogen-fixing bacteria of the soil environment, their role in breast health is not yet clear. Interestingly, studies have reported the presence of *Bradyrhizobium* in colitis patients, and in lower respiratory airways^39,40^. The TPBC tumor samples showed a significant increase in abundance of *Fusobacteria* and *Streptococcus* in TPBC samples, which is consistent with earlier work on *Fusobacteria* where it was shown to be enriched in malignant breast and colorectal cancer^12^. The genus *Streptococcus* contains both beneficial and pathogenic species: *S. thermophilus* is a beneficial species shown to be enriched in normal breast tissue and protective against DNA damage by its unique SOD activity^11^. However, at least 12 species have been shown to be pathogenic, of which two well-known species *S. pyogenes* and *S. pneumonia* have been found to be more abundant in breast tumor tissue than in the normal tissue^10^, and have also been found in human milk^42^.

One of the main objectives of this study was to identify if there are any race-specific microbes that could provide evidence for microbiome-host interactions in breast cancer pathogenesis as possible causes of health disparities, potentially leading to the development of more appropriate treatment options and possible preventive measures. The significance of higher abundance of the genus *Phenyllobacterium* in both WNH normal and tumor tissues is not clear. A recent study has shown that the higher abundance of this genus was observed in individuals with heavy alcohol drinkers with no sign of liver cirrhosis in comparison to non-alcohol drinkers^43^, suggesting a possible protective effect. A facultative bacteria, *Phenylobacterium zucineum* had been identified from human erythroleukemia cell line^44^, although its stable association with cell line is unclear. The significance of higher abundance of the genus of unclassified Bradyrhizobiaceae identified in BNH normal as compared to tumor is not clear. In addition, the reversal of abundance levels in WNH NAT vs tumor cohort need further studies. These observations suggest a possible role of host genetics and microbiota in the etiology of cancer. Members of the genus *Bradyrhizobium* are also one of the more commonly found members in human milk, and in select human tissues, in addition to soil^42^. However, that study did not describe the racial identity of the sample. Our study showed the abundance of the unclassified Caulobactereaceae is very low in both the NAT and the tumor tissue of BNH TNBC cohort. In contrast, both tumor and normal tissue of WNH cohort showed a higher abundance of this bacterial signature. The importance of the low abundance of this taxon in BNH TNBC racial breast cancer disparity is currently unclear. An earlier study on breast microbiome using Pathochip showed that the genus *Caulobacter* signature in tumor tissues of three subclasses of breast cancer including TNBC, and TPBC^15^. The genus *Phyllobacterium* showed a significantly higher abundance of approximately fourfold in BNH TPBC tumor tissue as compared to TPBC WNH tumor tissue. On the other hand, the abundance in WNH normal tissue exhibited very high interindividual variability, while in tumor its abundance was lower overall between the samples, suggesting that this genus could have a mechanistic link with BNH TPBC tumorigenesis, but an unclear role in WNH tumorigenesis. The genus *Phyllobacterium* has also been shown to increase in abundance in gastric adenocarcinoma and in sub-populations of patient samples with gut necrotizing enterocolitis, although a direct functional role has not been established^45,46^. The genus *Veillonella*, one of the well-known core members of the human breast milk microbiota, exhibited medium abundance in TPBC BNH tumor tissue as compared to normal, or to tumor tissue of the WNH group. However, a high interindividual heterogeneity was also evident among BNH TPBC group. Interestingly, a recent study showed that distribution of this genus showed a higher abundance in infant fecal samples from African countries (30 to 50%) as compared to infant fecal samples from the US (20–30%)^47^. *Veillonella*^48^ has also been found to be one of the earliest sacchharolytic colonizers of the developing infant as a minor core member (5%). It is also known to interact with other core microbial members including *Streptococcus* and *Bifidobacterium* to produce the key anti-inflammatory short chain fatty acid propionate, and is known to be one of the main members of healthy oral and gut microbiota^48^.

One of the limitations of this study is that only a limited number of TPBC-BNH samples were used. The significantly higher abundance in BNH TPBC as compared to WNH TPBC observed here should be verified with a larger scale sampling. The significantly higher abundance of the phylum Thermi was accompanied with higher interindividual variability in NAT as compared to tumor tissue of BNH TNBC cohort. In contrast, WNH TNBC showed an opposite abundance profile, with low in normal and high in tumor tissue. In another published study, Thermi was observed in lung adenocarcinoma patients with increased abundance in late stages of tumor^49^. Considering the environmental resistance traits of some members of the phylum, it is possible that this microbe acts to protect the host from insults, while the relative changes in abundance over threshold levels may indicate unfavorable host response to the disease state. On the other hand, the role played by the microbe may be race-specific as the WNH TNBC exhibits an opposite trend shown for lung tumors^49^.

A thorough understanding of the breast microbiome as impacted by host genetics, life-style, socioeconomic factors, and geography will be necessary to identify both the beneficial and harmful microbiota that may influence breast cancer development. Deeper understanding of these factors may lead to development of microbiome-based therapeutic strategy for breast cancer treatment.

## Materials and methods

### Study population and sample collection

The frozen breast tissue samples collected from the biorepository were collected and processed in accordance with the UNTHSC IRB protocol (#2009–001). The deidentified, frozen breast tissue samples (n = 50) were acquired from the biorepository at Fox Chase Cancer Center, Philadelphia, PA (additional information is described in results section). For the first set of microbiome analysis, we included 8 White non-Hispanic, of which 2 patient samples (n = 4; n = 2 tumor and n = 2 NAT)) were excluded from final analysis due to admixed ancestry, 7 Black non-Hispanic women with a triple negative breast cancer (TNBC), 7 White non-Hispanic triple positive breast cancer (TPBC), and 3 Black non-Hispanic women with TPBC. For the second set of microbiome analyses, we included, 10 additional White non-Hispanic WNH tumor and matched Normal Adjacent to Tumor (NAT) tissues (described in results section). The matched NAT from the same individuals were considered as normal. The patient’s information on race, ethnicity, age at diagnosis, pathology report on stage of tumor as T1, T2 and T3 were collected on the samples (Table S1). All the tissue samples were derived from women subjects diagnosed with breast cancer. The biorepository information revealed that patients were not treated with antibiotics prior to the tissue collection although duration window of non-exposure to antibiotics prior to surgery was not included in their data (Table S1). The specimens are classified as BNH and WNH according to the self-reported data of patients from their respective geographical areas.

### Ancestry analysis of breast tissue

The genomic DNA isolated for microbiome analysis was used for genome-wide SNP profiling to verify the racial identity of patient samples (Fig. 1**)**. Admixture analysis for ancestry assessment was performed on genomic SNP data obtained from the Infinium Global Screening Array (Illumina). Data were analyzed by the method described previously^50^, and were pruned based on linkage disequilibrium. SNP genotypes downloaded from the Human Genome Diversity Project for the European, African, Americas/Amerindian and Asian super populations were merged with the sample dataset. Ancestry coefficients for the four parent ancestral populations (k = 4) were estimated for based on LD-pruned nDNA SNPs using an unsupervised model-based approach as implemented in ADMIXTURE^51^.

### 16S rRNA library preparation and Illumina MiSeq sequencing

Generation of the 16S rDNA library, sequencing, raw read calling/processing, were performed by Zymo Research through their ZymoBIOMICS service (Zymo Research Corp. Irvine, CA). ZymoBIOMICS Microbial Community Standards (Zymo D6300) were used as positive controls. The details of the methods used is as follows: Genomic DNA was extracted from frozen breast tissue using the ZymoBIOMICS 96-bead DNA Extraction Kit (Zymo Research Corp). 16S rRNA gene amplicon library was prepared using Quick-16 NGS Library Prep Kit (Zymo Research Corp.). Briefly, 16S rRNA gene was amplified with Illumina MiSeq compatible Quick-16 Primer Set 341F (CCTACGGGNGGCWGCAG) and 785R (GACTACHVGGGTATCTAATCC) targeting the V3-V4 hypervariable region. The PCR master mix containing: 10 μl of Quick-16S qPCR Premix, 4 μl of Quick-16S Primer Set V3–V4, 4 μl of ZymoBIOMICS DNase/RNase Free Water, and 2 μl DNA template. Both positive and negative controls for PCR quality assessment were included during amplification. The majority of samples were amplified for 18 cycles except low microbial DNA concentration samples were amplified for an additional 4 cycles. The thermocycler conditions were: 95 °C for 10 min, 20 cycles of 95 °C for 30 s, 55 °C for 30 s, 72 °C for 3 min and hold temperature, of 4 °C. Following amplification, estimation of DNA quality and concentration, followed by enzymatic clean-up was performed and samples were stored at 4 °C prior to sequencing.

Specific barcodes were added for each sample through index PCR amplification. The 20 μl PCR reaction recipe composed of 10 μl Quick-16S qPCR Premix, 4 μl ZymoBIOMICS DNase/RNase Free Water, 2 μl each of the Index Primer set A, and 2 μl purified DNA. The PCR condition was: 95 °C for 10 min, 5 cycles of 95 °C for 30 s, 55 °C for 30 s, 72 °C for 3 min followed by holding temperature at 4 °C. The amplified library was quantified and stored at 4 °C. A fluorescence standard curve was created by running in parallel real-time PCR reaction with the same thermocycler program as above using 20 μl with fluorescence standard, in triplicate configuration. Equal volumes of bar-coded samples were pooled prior to purification of the library. The pooled library was purified using Select-a-Size Zymo MagBeads (Zymo Research Corp.) following manufacturer’s specifications. The purified index bar-coded library was then denatured, diluted, and sequenced on Illumina MiSeq.

### Bioinformatics analysis

The raw sequence data were analyzed using mothur (v.1.36.1) program following the MiSeq SOP as previously described^52^. Briefly, contigs were assembled from paired-end raw reads sequences with removal of ambiguous sequences from the dataset. Sequences were dereplicated and aligned against the SILVA 16S rRNA database (v132). Gaps were deleted from the aligned contigs following deletion of unaligned sequence reads with database reference sequences. The sequences were further dereplicated using Unique seqs and Precluster Commands and chimeras were identified and removed using UCHIME algorithm^53^. Operational taxonomic units (OTUs) were grouped based on 97% sequence similarity using the average neighbor clustering algorithm^54^. Taxonomic assignment was determined by comparing with the Green Genes database using RDP classifier with a minimum of 80% confidence^55^. Non-bacterial sequences including mitochondria, chloroplast, archaea and eukaryote, as well as undefinable sequences were excluded from the data set. Microbial community diversity (Shannon diversity), evenness and richness (Chao1 and abundance coverage-based estimator (ACE) were calculated at 97% cut-off after rarefied to the lowest sampling depths of 1,238 reads per sample^56–58^. Principle Coordinate Analysis (PCoA) based on Uni-Frac distances were performed to compare the microbial community differences among different samples^59^. The Molecular Variance analysis (AMOVA) was performed to assess the variability among and within different groups^60^. We also compared the microbial fingerprints differences between normal and tumor tissue as well as between different ethnic groups at phylum level and genus level. Abundant taxa (bacterial phyla > 1% and genera > 5%) were selected for this analysis. Wilcoxon Rank Sum Test or Wilcoxon Signed Rank Test were used to screen the significantly different bacterial taxa. Wilcoxon Rank Sum Test or Wilcoxon Signed Rank Test were performed using R program.

## Supplementary information

Supplementary Table S1.

Supplementary Table S2.

## Data Availability

The data used in this study has been deposited in NCBI-SRA database. The raw sequence reads generated for this study are available in the NCBI Sequence Read Archive (SRA) under the BioProject ID PRJNA637875.
