# Supplementary material for: Comparative analysis of racial differences in breast tumor microbiome
Source: Sci Rep. 2020 Aug 24;10:14116. doi: 10.1038/s41598-020-71102-x (PMC7445256; doi:10.1038/s41598-020-71102-x)
Supplement: Supplementary file 1 — Supplementary Table S1. [file 41598_2020_71102_MOESM1_ESM.docx]

**Comparative Analysis of Racial Differences in Breast Tumor Microbiome** *Srikantha Thyagarajan^1,2^, Yan Zhang^1^, Santosh Thapa^3,4^, Michael S. Allen^1^, Nicole Phillips^1^, Pankaj Chaudhary,^,2,^ Meghana V. Kashyap^5^ and Jamboor K. Vishwanatha^1,2^**

^1^Department of Microbiology, Immunology, and Genetics, University of North Texas Health Science Center, Fort Worth, Texas 76107 USA

^2^Texas Center for Health Disparities, University of North Texas Health Science Center, Fort Worth, Texas 76107 USA

^3^Texas Children's Microbiome Center, Department of Pathology, Texas Children's Hospital, Houston, Texas, 77030 USA

^4^Department of Pathology and Immunology, Baylor College of Medicine, Houston, Texas, USA

^5^University of Nebraska Medical Center, Omaha, Nebraska, USA

* Corresponding Author:

**Table S1.** Characteristics of the women breast cancer patient samples used in this study. Ethnicity is reported as non-Spanish, non-Hispanic for all groups in the biorepository. In this study, White non-Hispanic and Black non-Hispanic refers respectively to WNH and BNH in the text. Racial groups, Caucasian and African, refers to the self-reported information as white or black in the repository. Pathologic Stage group indicates the tumor as assessed by the pathological report using TNM staging system and the total number is indicated (n) for each tumor stage group.

| Sample | Tissue Type | Ethnicity | Race | Tumor Type | Total Number | Pathologic Stage group | Age range |
| --- | --- | --- | --- | --- | --- | --- | --- |
| a set |  |  |  |  |  |  |  |
| WNHa_1t-6t | Tumor | White non-Hispanic | Caucasian | TNBC | n=6 | T2=3 and T3=2 and 88=1 | 33-78 |
| WNHa_9n-14n | Matched- normal | White non-Hispanic | Caucasian | TNBC | n=6 | T2=3 and T3=2 and 88=1 | 33-78 |
| BNHa_17t-23t | Tumor | Black non-Hispanic | African | TNBC | n=7 | T1=3, T2=2,T3=2 | 27-66 |
| BNHa_24n-30n | Matched-normal | Black non-Hispanic | African | TNBC | n=7 | T1=3, T2=2,T3=2 | 27-66 |
| WNHa_31t-37t | Tumor | White non-Hispanic | Caucasian | TPBC | n=7 | T2=6,T3=1 | 41-68 |
| WNHa_38n-44n | Matched- normal | White non-Hispanic | Caucasian | TPBC | n=7 | T2=6,T3=1 | 41-68 |
| BNHa_45t-47t | Tumor | Black non-Hispanic | African | TPBC | n=3 | T1=1,T2=1 T3=1 | 25, 49, 68 |
| BNHa_48n-50n | Matched- normal | Black non-Hispanic | African | TPBC | n=3 | T1=1,T2=1 T3=2 | 25, 49, 68 |
| b set |  |  |  |  |  |  |  |
| WNHb_1t-10t | Tumor | White non-Hispanic | Caucasian | TNBC | n=10 | T1=2; T2=6; T3=2 | 31-57 |
| WNHb_13n-22n | Matched- normal | White non-Hispanic | Caucasian | TNBC | n=10 |  | 31-57 |
